# Unusual Bioactive Compounds with Antioxidant Properties in Adjuvant Therapy Supporting Cognition Impairment in Age-Related Neurodegenerative Disorders

**DOI:** 10.3390/ijms221910707

**Published:** 2021-10-02

**Authors:** Natalia Cichon, Angela Dziedzic, Leslaw Gorniak, Elzbieta Miller, Michal Bijak, Michal Starosta, Joanna Saluk-Bijak

**Affiliations:** 1 Biohazard Prevention Centre, Faculty of Biology and Environmental Protection, University of Lodz, Pomorska 141/143, 90-236 Lodz, Poland; natalia.cichon@biol.uni.lodz.pl (N.C.); leslaw.gorniak@biol.uni.lodz.pl (L.G.); michal.bijak@biol.uni.lodz.pl (M.B.); 2 Department of General Biochemistry, Faculty of Biology and Environmental Protection, University of Lodz, Pomorska 141/143, 90-236 Lodz, Poland; angela.dziedzic@edu.uni.lodz.pl; 3 Department of Neurological Rehabilitation, Medical University of Lodz, Milionowa 14, 93-113 Lodz, Poland; elzbieta.dorota.miller@umed.lodz.pl (E.M.); michal.starosta@umed.lodz.pl (M.S.)

**Keywords:** antioxidants, cognition impairment, diet patterns, age-related neurodegenerative disease, dementia, flavonoids, melatonin, propolis, sulforaphane, *N*-acetylcysteine

## Abstract

Cognitive function decline is strictly related to age, resulting in the loss of the ability to perform daily behaviors and is a fundamental clinical neurodegeneration symptom. It has been proven that an adequate diet, comprehensive nutrition, and a healthy lifestyle may significantly inhibit neurodegenerative processes, improving cognitive functions. Therefore, intensive research has been conducted on cognitive-enhancing treatment for many years, especially with substances of natural origin. There are several intervention programs aimed at improving cognitive functions in elderly adults. Cognitive functions depend on body weight, food consumed daily, the quality of the intestinal microflora, and the supplements used. The effectiveness in the prevention of dementia is particularly high before the onset of the first symptoms. The impact of diet and nutrition on age-associated cognitive decline is becoming a growing field as a vital factor that may be easily modified, and the effects may be observed on an ongoing basis. The paper presents a review of the latest preclinical and clinical studies on the influence of natural antioxidants on cognitive functions, with particular emphasis on neurodegenerative diseases. Nevertheless, despite the promising research results in animal models, the clinical application of natural compounds will only be possible after solving a few challenges.

## 1. Introduction

The global growth of neurodegenerative disorders affecting elderly adults will soon become a worldwide health problem. In the last century, the disturbing elevation in the size of the aging population has translated into globally superior rates of dementia and age-related cognitive decline [1]. Age is a relevant determinant of cognitive impairment; however, other contributing factors, including demographic, environmental, genetic, lifestyle, and nutrition, also have a tremendous impact [2]. Age-related neurodegenerative diseases are a broad range of neurological disorders affecting separate subsets of neurons in particular anatomic systems. The most common age-related neurodegenerative diseases are Alzheimer’s disease (AD), Parkinson’s disease (PD), Huntington’s disease, and late-onset of multiple sclerosis (LOMS) [3,4]. Extrapyramidal and pyramidal movement deviation and cognitive or behavioral irregularity are typical symptoms of neurodegenerative diseases [5]. 

Cognitive impairment is a fundamental clinical symptom in neurodegeneration and arises from progressive brain damage, mainly in the hippocampus and cerebral cortex, resulting from the long-term neuroinflammatory process. Cognitive functioning is divided into the following main domains: (1) learning and memory, (2) language, (3) visuospatial, (4) executive, and (5) psychomotor. Cognitive impaired are diagnosed as mild (MCI) or significant based on the severity of their symptoms (known as dementia) [6]. The elementary cognitive abilities include perception, feeling emotions, memory (declarative, episodic, and semantic), and orientation, whereas the complex ones comprise abstract thinking, imagination, as well as verbal, visual-spatial, and executive functions [7]. Cognitive dysfunctions also include aphasia, apraxia, and impaired judgment. There may also be behavioral/personality disturbances, including psychosis, depression, or agitation [8].

Aging-related neurodegenerative diseases can be due to the absence of protective mechanisms caused by dietary deficiencies and a small supply of antioxidants. Therefore, higher consumption of antioxidants may inhibit the devastating effects of reactive oxygen species (ROS) on neurons and hence protect against neurodegenerative diseases, such as dementia. In the pathogenesis of neurodegenerative diseases and dementia, oxidative stress is strongly implicated [9]. Epidemiological studies have shown that cognitive impairment often co-exists with elevated oxidative stress and inflammation parameters [10,11]. The brain is susceptible to oxidative damage because it has a high level of fatty acids, enhanced oxygen consumption, and a relatively low level of antioxidants [12]. Furthermore, the accumulation of free radicals in the brain increases a blood-brain barrier (BBB) permeability, thus causing neuroinflammation and neuronal loss [13]. Chronic oxidative stress may induce cellular damage, impair the DNA repair system, and mitochondrial dysfunction, all of which have been known as critical factors in accelerating of the aging process and developing of neurodegenerative disorders [14]. Nitric oxide synthase (iNOS) is a significant contributor to initiation/intensification of the CNS inflammatory and neurodegenerative conditions through the excessive production of nitric oxide (NO), which generates ROS and reactive nitrogen species (RNS). Therefore, activation of iNOS and NO generation has come to be accepted as a marker and therapeutic target in neuroinflammatory conditions [15]. Moreover, oxidative stress disrupts the insulin-dependent signaling pathway and may affect the increased production of interleukin (IL) 6, thus worsening neurons’ efficiency and leading to neuronal death [16]. 

Limited blood supply to the brain is also an essential contributor to cognitive decline. In elderly, an imbalance between pro- and anti-coagulant processes is widely observed [17]. Altered vascular function is mainly caused by impaired endothelial function and is particularly prominent in elderly people with evidence of cardiovascular disease, obesity, and diabetes [18]. Decreased cerebral blood flow and disruption of blood circulation of some brain regions, causes undersupply of oxygen (hypoxia) leading to inflammatory and neurodegenerative alterations in the brain [19]. 

**Figure 1:**
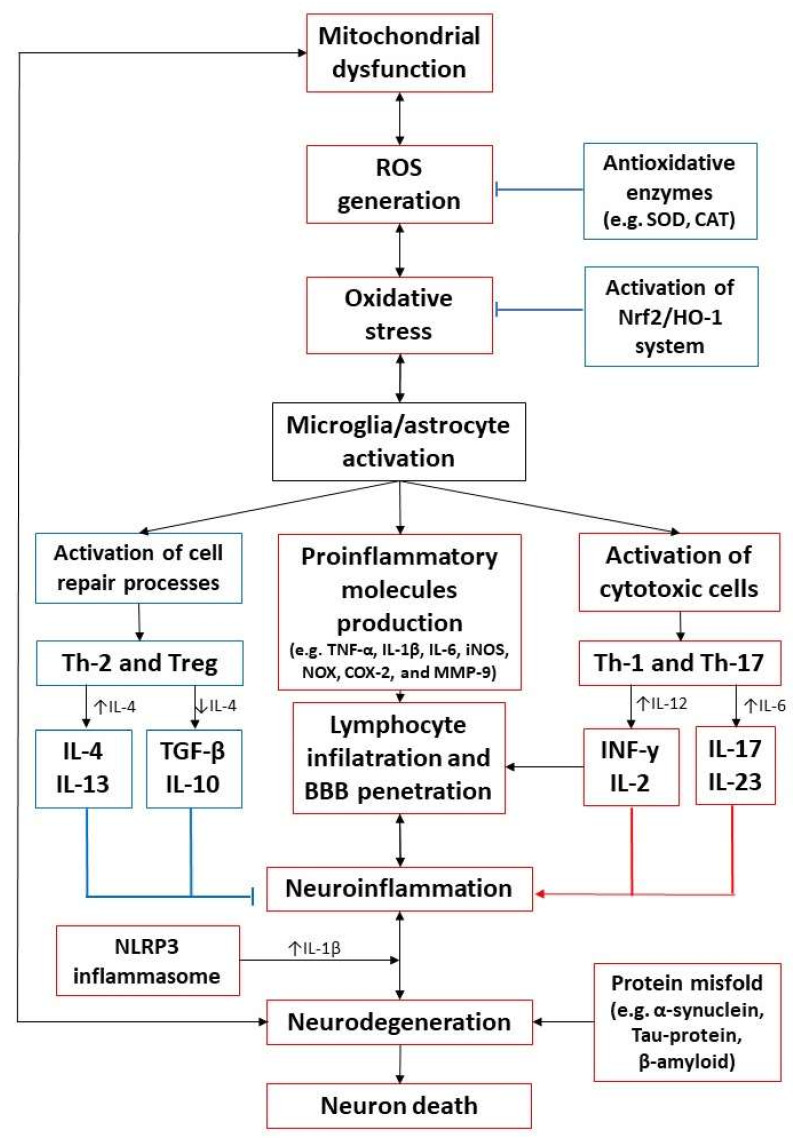
Oxidative and inflammation pathways in neurodegeneration. Increased ROS production caused, inter alia, by mitochondrial dysfunction, generating oxidative stress, is a direct reason for neuroinflammation and neurodegeneration. ROS stimulates altered intracellular signaling leading to microglia and astrocyte activation, characterized by a dual response depending on the activation time. In the acute phase of damage, cell repair processes are implicated; however, the persistent activation state leads to their overactivation and, consequently, the secretion of pro-inflammatory mediators, BBB damage, and T-cell infiltration. Thus, chronic inflammation causes neurodegeneration expressed by neuronal damage and death. CAT–catalase; COX-2–cyclooxygenase-2; iNOS–inducible nitric oxide synthase; INF-γ–interferon y; IL–interleukin; MMP-9–matrix metallopeptidase 9; NOX–NADPH oxidase; Nrf2/HO-1-nuclear factor erythroid 2-related factor/Hemoxygenase 1; ROS–reactive oxygen species; TGF-β–transforming growth factor β; Th–T helper cell; TNF-α–tumor necrosis factor α; Treg–T regulatory cell; SOD–superoxide dismutase.

In addition, the aging process is related to the alterations in secretory patterns of the hormones by the endocrine system, produced mainly by the hypothalamic-pituitary axis modification. Therefore, the levels of neurotransmitters and neurohormones also decrease with age. Moreover, the co-existence of various diseases may cause secondary changes in the levels of hormones and enzymes [20]. The oxidative and inflammatory pathways potentially contributing to the progression of neurodegenerative disease are summarized in Figure 1.

## 2. Antioxidant-Rich Diet on Age-Related Cognitive Impairment 

A diet rich in antioxidants may partially contribute to the alleviation of cognitive disorders resulting from the course of neurodegenerative diseases. Recent studies imply that the use of specific diets rich in antioxidants and anti-inflammatory components, together with reduced caloric intake or use of caloric restriction mimetics, may lower age-related cognitive declines, reduce the risk of cardiovascular disease, and risk of developing the neurodegenerative disease [21,22]. Thus, it has been suggested that an adequate supply of nutrients, together with proper and controlled supplementation, can notably slow the aging brain, feasibly leading to improved cognition and motor abilities, with these phenomena likely having a bidirectional effect [23]. However, special attention should be taken to calorie restriction in elderly people to avoid malnutrition and unintentional weight loss [24]. In the elderly with clinically significant cognitive impairment, problems with food intake are often observed, consequently causing malnutrition, which implies the progression of the disorder at every stage [25]. In the advanced stage of dementia, these phenomena are even more pronounced [26]. 

The effect of diet and nutrition on age-related cognitive impairment is becoming a growing branch as a potential modulatory contributor [27]. In pre-clinical studies conducted on animal models, it has been shown that administering compounds facilitating the synthesis of phospholipids in cell membranes implies an increase in the concentration of specific synaptic proteins, thus leading to synaptogenesis [28]. Moreover, it has been proposed that the appropriate combination of these nutrients increases dendrites, which are an anatomical marker of new synapses responsible for improving cognitive functions [29]. Multiannual observation of behavior and eating habits in large populations, as well as indicators of adherence to specific dietary guidelines, allow a conclusion about the protective effect of diet as a potentially modifiable element of lifestyle. Scientists thus hope that both dementia risk and progress are modifiable. [30]. Varied dietary patterns have been studied in association with their pro-healthy impact on cognitive functions, demonstrating that the benefit of nutritional factors may derive from synergistic interactions of distinct components contained in a specific food pattern [31]. 

It is crucial to establish an effective way to enhance healthy aging and delay age-related diseases. There is still a lack of effective pharmaceutical treatments, which will prevent cognitive decline in neurodegenerative diseases. There is substantial evidence confirming an association between diet and cognitive functions; therefore, nutritional approaches to avert or slow cognitive impairment could have an extraordinary health impact. Moreover, further research is necessary to understand the potential protective effects of antioxidants on the course of neuroinflammatory diseases. Such studies will improve understanding of the disease’s mechanisms, help implement proper dietary prevention, and establish new goals for innovative treatments that provide real therapeutic benefits in CNS diseases.

## 3. In Vivo Studies and Clinical Trials on Improving Cognition by Various Antioxidants

Based on in vivo studies, it has been proved that the mechanism of action of antioxidants is related to the modulation of cell signaling pathways associated with cognitive processes; stimulation of synaptic plasticity; participation in the expression of genes encoding antioxidant enzymes, and neurotrophic factors; as well as improvement of cerebral circulation [32,33,34]. Among the many bioactive phytocompounds described so far, we chose those that have the best documented health-promoting properties in many aspects of health, including, in part, suppressing cognitive disorders and the molecular mechanisms of their action are relatively well understood. It seems that the most promising pro-cognition compounds are flavonoids–baicalin, quercetin, and epigallocatechin gallate (EGCG). In addition to supplementation with single flavonoids compounds, it is worth paying attention to other unusual bioactive compounds, such as propolis, melatonin, sulforaphane, and N-acetylcysteine (NAC).

### 3.1. Studies on Animal Models

Recent studies have demonstrated that selected natural compounds are effective in delaying age-related deficits in motor functions and spatial memory and improve short-term memory and learning [35,36]. Summarizing of the current preclinical in vivo studies, this section examines the impact of selected natural antioxidants on cognitive functions that can potentially be used clinically.

#### 3.1.1. Baicalin

Baicalin (5, 6-dihydroxy-7-O-glucuronide flavone) is a flavonoid in *Scutellaria baicalensis*, with anti-oxidant, anti-inflammatory, anti-apoptotic, and anti-coagulant properties [37]. Baicalin is poorly absorbed in the gastrointestinal tract due to its polar structure preventing passive diffusion through the lipid bilayer, in contrast with baicalein, which is highly lipophilic and has a good absorption profile throughout the gastrointestinal tract. It has been shown that baicalein is the more preferred form for oral absorption, while baicalein is first hydrolyzed to baicalein by the gut microbiota or enzymes, lactase-phlorizin hydrolase or beta-glycosidase, before being absorbed in the gut. After rapid absorption into the plasma, baicalin becomes bound to proteins, mainly human serum albumin (HSA) [38]. Based on studies using reversed-phase HPLC, it has been observed that baicalin penetrates easily through the BBB, with the highest concentrations observed in the hippocampus, striatum and thalamus [39]. It has been shown that baicalin alleviates cognitive impairment in experimental animal models [40,41,42]. Baicalin may also prevent neuronal loss induced by amyloid β (Aβ) peptide, widely regarded as the major player in AD pathogenesis. Accumulation of Aβ oligomer may stimulate endoplasmic reticulum stress-induced apoptosis [43]. In addition, it has a neuroprotective function against ischemia-reperfusion injury through activation of γ-aminobutyric acid (GABA) signaling [44], diminished inflammatory activation of microglia [45], and reduced hippocampal neuronal damage through the inhibition of matrix metalloproteinase 9 (MMP-9) activity [46]. Under physiological conditions, pro-inflammatory cytokines, including TNF-α and IL-6, are not present or expressed very low in the brain. However, they can be induced by Aβ peptide in microglia, astrocytes, neurons, and endothelial cells, causing neurodegeneration [47]. Chen et al. have reported that 100 mg/kg of baicalin treatment may effectively improve memory deficits, reduce glial cell activation, and diminish the level of IL-6 and TNF-α in Aβ-injected ICR mice (a strain of albino mice originating in Swiss, named after the initial letters of the Institute of Cancer Research) in comparison to the control group [48]. Furthermore, Jin et al. have observed that mice treated baicalin (100 mg/kg for 2 weeks) revealed a reduced microglia activation, neuronal apoptosis, and reduced levels of pro-inflammatory cytokines by inhibiting the TLR4 (toll-like receptor 4)/NF-κB (nuclear factor kappa-light-chain-enhancer of activated B cells) pathway and NLRP3 (nucleotide-binding domain (NOD)-like receptor protein 3) inflammasomes [40]. The NLRP3 inflammasome is a multimeric protein complex that initiates an inflammatory form of cell death and triggers the release of such pro-inflammatory cytokines as IL-1β and IL-18 [49]. The NLRP3 inflammasome has been implicated in a broad range of neurodegenerative diseases [50]. In another study, Ma et al. assessed the effect of baicalin (50-200 mg/kg for 7 weeks) on diabetes-related cognitive deficits in rats. Baicalin has been shown to reverse cognitive impairment in diabetic rats and significantly enhances neuronal survival [51]. It also exhibits an ability to regulate the level of mitogen-activated protein kinases (MAPKs) by enhancing the extracellular signal-regulated protein kinase (ERK) level and reducing the level of a critical player in the production of pro-inflammatory cytokines: JNK (c-Jun NH2-terminal kinase) and p38 [52]. The neurorestorative properties of baicalin are associated with regulation of mitochondrial function and suppression of Ca2+/calmodulin (CaM)-dependent protein kinase II (CaMKII) phosphorylation [53], known to have a fundamental role in synaptic plasticity and memory formation [54]. Moreover, it inhibits apoptosis and promotes neuron proliferation by modulation of glycogen synthase kinase 3 (GSK3b), Akt, and angiopoietin 1 (Ang-1) [55]. Wang et al. have demonstrated that Aβ-injected Wistar rats fed baicalin (50 and 100 mg/kg per day, 20-day treatment) attenuated apoptosis comparison to control animals also Aβ-injected rats, but not fed baicalin. The anti-apoptotic effect of baicalin is based on the modulation of the expression of genes related to apoptosis (Bax, Bcl-2, caspase-3, and cytochrome c) [56]. 

#### 3.1.2. Quercetin

Quercetin (3,5,7,3′,4′-pentahydroxyflavone) belongs to the flavonoid group abundantly present in apples, honey, raspberries, onions, red grapes, cherries, citrus fruits, and green leafy vegetables [57]. Quercetin aglicon is passively absorbed in the small intestine, while its glycosides are first deglycosylated by enzymes of the intestinal microflora (lactase-florin hydrolase and/or beta-glucosidase) [58,59]. Subsequently, quercetin aglycone is converted to methylated, sulfate and glucuronidated metabolites. Based on animal studies, quercetin has been shown to cross the BBB, however, its bioavailability is low, pico-nanomolar concentration [60]. Nevertheless, intensive research is being conducted to increase the bioavailability of quercetin [61]. Sriraksa et al. have demonstrated that quercetin mitigates the neurotoxicity and cognitive impairment in adult male Wistar rats injected by 6-hydroxydopamine (6-OHDA) (a neurotoxin mimic Parkinsonism in rodents) [62,63]. Quercetin, at all doses (100, 200, and 300 mg/kg) has shown a beneficial effect on memory and learning in 6-OHDA rats, compared with wild-type rats [63]. Notably, only quercetin in a high dose (300 mg/kg) decreased acetylcholinesterase (AChE) activity [63], increasing available acetylcholine. This essential neurotransmitter plays a vital role in the learning and memory process [64]. They have also shown that quercetin increases neuron density estimated in the hippocampal homogenate from 6-OHDA injected rats, compared with control rats [63], which, probably, leads to neurodegeneration process inhibition [65]. Interestingly, in the same study, it was reported that the rats subjected to the high dose of quercetin (300 mg/kg) had a meaningfully increased activity of the scavenging enzymes superoxide dismutase (SOD), catalase (CAT), and glutathione peroxidase (GPx) in the hippocampus, compared withwith control rats who not treated with any dose of quercetin [63]. It is proposed that the cognitive-enhancing effect of quercetin might be due to its anti-oxidant effect by promoting the activities of scavenging enzymes to protect neurons from oxidative injury, supporting the survival of neurons in the hippocampus [66]. Furthermore, Wang et al. have reported that quercetin (40 mg/kg, for 16 weeks) was known to improve learning and recognition, as well as reduce mitochondrial dysfunction, as indicated by growing mitochondrial membrane potential, ATP level, and AMP-activated protein kinase (AMPK) activity, and diminish free radical generation, in a mouse model of AD (the APP^swe^/PS1dE9 transgenic mice) [67]. Another animal study has demonstrated that oral administration of quercetin (60 mg/kg, for 16 weeks) in high cholesterol-fed aged mice inhibits the cholesterol-stimulated activation of protein phosphatase 2C alpha and activates AMPK [68]. Quercetin also reduces the level of inflammatory biomarkers through the blockage of NF-κB/p65 nuclear translocation, improves cognitive functioning, and diminished the expression of β-amyloid converting enzyme 1, resulting in a reduction of the Aβ deposits [68]. Moreover, quercetin can inhibit cytokine and iNOS expression by the inhibition of the NF-κB pathway both in vitro and in vivo [69,70]. Other studies have reported that quercetin supplementation significantly increased learning and ameliorated memory impairment [71], as well as improves memory recall [72] in an animal model of AD. 

#### 3.1.3. Epigallocatechin Gallate

Green tea and its main polyphenolic compound, EGCG, have been proposed to exhibit neuroprotective effects on animal models. Levites et al. have demonstrated that EGCG shows a neuroprotective effect in the *N*-methyl-4-phenyl-1,2,3,6-tetrahydropyridine-induced mouse model of PD [73]. EGCG has low oral bioavailability (0.1–0.3%), is poorly absorbed by the body, reaches micromolar concentrations in plasma that are detectable in the plasma for several hours (<8 h). This flavonoid is hydrolyzed by the intestinal microbiota to produce the metabolites gallic acid (GA), and (-)-epigallocatechin (EGC), which are present in the plasma in their conjugated and free forms and are characterized by higher bioavailability than EGCG [74]. Importantly, it has been shown that both EGCG and its metabolites, at low concentrations (0.05 μM), penetrate the BBB, reaching the brain parenchyma and inducing neurogenesis [75,76]. It has been reported that EGCG treatment significantly improves cognitive deficits, amyloid precursor protein processing, and tau pathology in D-gal-induced AD mice, Tg2576 (APP^swe^), and PS2 transgenic mice [77,78]. Wei et al. have shown that intragastric administration of EGCG (100 mg/kg for 4 weeks) significantly reduces BBB permeability and improves learning and memory in rats with cognitive dysfunction, thus providing solid evidence that EGCG may be a supplementary compound in the anti-neurodegeneration treatment [79]. Furthermore, EGCG inhibits the release of proinflammatory cytokines, including TNF-α, IL-1β, IL-6, and soluble intercellular adhesion molecule-1 (ICAM-1) in lipopolysaccharide (LPS)-induced macrophages and neurons and diminishes the production of ROS in vitro [80]. The effect of long-term oral administration of polyphenon E (green tea extract) mixed with water on young rats’ spatial cognition learning ability has been shown. Relative to controls, rats administered polyphenon E (in concentrations: 0.1% and 0.5%) improved reference and working memory-related learning abilities and had lower plasma concentrations of lipid peroxides and higher plasma ferric-reducing anti-oxidation power. Moreover, rats supplemented by polyphenon E had inferior hippocampus ROS concentrations compared with those in control rats [81].

#### 3.1.4. Propolis 

Propolis, known as bee glue, is a natural resinous mixture produced by honeybees from substances collected from parts of plants, buds, and exudates [51]. Of note, the main biologically active components of propolis are flavonoids (flavones and flavanones), phenolic acids (such as cinnamic acid, galangin, crysin, pinostrobin, pinobanksin, and pinocembrin), and their esters [82]. Pinobanksin and pinocembrin are the most abundant (70%) flavonoids in propolis [83]. Due to the multitude of substances contained in propolis, it is suggested that its neurorestorative effect is expressed both through the direct influence of the brain, via blood circulation, and through the gut-brain axis (GBA) [84]. Pinocembrin (5,7-dihydroxyflavanone) is a natural product extracted from propolis and can protect against cerebral ischemia by providing neuroprotection and improving neurovascular unit function [85]. Interestingly, pinocembrin can pass through the BBB through a passive transport process conducted by P-glycoprotein (membrane transporter), present in endothelial cells [86]. Pinocembrin also restrains neuroinflammation by downregulating the receptor for advanced glycation end-products [87]. Meng et al. have demonstrated that pinocembrin treatment alleviates cognitive impairments, diminishes neurological scores, declines neuronal loss in the hippocampus, and decreases the level of glial fibrillary acidic protein (GFAP)-positive cells in the hippocampal CA1 region in a rat model of transient global cerebral ischemia [88]. Furthermore, pinocembrin has noticeably enhanced neuronal survival in the frontal cortex and hippocampal CA3 region in diabetic encephalopathy mice [89]. Another flavonoid occurring abundantly in propolis is pinobanksin. It has been reported that oral administration of pinobanksin (5 and 10 mg/kg for 5 weeks) dramatically improves the cognitive performance of rats with vascular dementia [90]. The protective effect of pinobanksin might be attributed to its intense antioxidant action, as evidenced by the remarkably decreased malondialdehyde (MDA) level, as well as the increased superoxide dismutase (SOD) activity and glutathione (GSH) level in rats fed with pinobanksin [90]. In addition, it seems that pinobanksin stabilizes the mitochondrial redox balance by scavenging ROS directly or diminishing the ROS formation by protecting the electron transfer chain [90].

Many studies have revealed that propolis displays protective abilities against neurodegenerative damage, related to cognitive impairment mainly via its antioxidant features. Propolis was also demonstrated to increase the expression of brain-derived neurotrophic factor (BDNF) and activity-regulated cytoskeleton-associated protein (Arc), being critical factors for synaptic conduction [91]. It has been confirmed by Nanaware et al. who have reported that oral administration of ethanolic extract of propolis (100, 200, and 300 mg/kg) significantly reverses the cognitive impairment of βA-induced rats, which, among other things, is associated with decreased MDA level (a marker of lipid peroxidation) in rat brains (*p* < 0.01) [92]. Additionally, the same study has reported propolis administration results in dose-dependent acetylcholinesterase inhibition, enhances brain monoamine level, and improves memory deficits (estimated by enhanced BDNF level), which suggests that many mechanisms might be involved in that neuroprotective action of Indian bee propolis [92]. In a kainic acid-induced excitotoxicity rat model, it was reported that propolis (75 and 150 mg/kg) attenuated NO, TNF-α, and caspase-3 levels in vivo and prevented neuronal loss in rat brains, and ameliorated seizures attacks [93]. 

A recent study has reported that Brazilian green propolis displays its neuroprotective effect through its antioxidant mechanism of action, which could be attributed to the synergistic effect of its main components, including caffeoylquinic acid derivates, artepillin C, and p-coumaric acid. These compounds have beneficial effects on healthy cognition, which has been proved by many in vitro and in vivo studies. In general, p-coumaric acid is shown to reduce oxidative stress, inhibit genotoxicity and exert neuroprotection. Moreover, p-coumaric acid improves cognitive problems caused by an abnormality of the cholinergic nervous system in rats [94]. Following compound, artepillin C prevents oxidative damage and suppresses lipid peroxidation in the HepG2 cells line [95]. At the same time, caffeoylquinic acid reduces Aβ deposition in the brain by modulating the Aβ clearance pathways, ameliorating cognitive decline and neuronal loss in APP/PS2 mice [96]. Wu at al. have also reported that Brazilian green propolis displays its inhibitory effect on oxidative stress, pro-inflammatory cytokines, and apoptosis and protection against neuronal damage through its anti-inflammatory, antioxidant, and anti-apoptotic properties [97].

#### 3.1.5. Melatonin

Melatonin (*N*-acetyl-5-methoxytryptamine) is an endogenous neurohormone derived from tryptophan that modulates a broad range of physiological processes, such as circadian rhythm and anxiety, immune response, and free radical scavenging [98,99]. Exogenously administered melatonin has a similar biological effect to endogenous. However, the method of melatonin administration, hepatic metabolism, and the individual absorption rate determine its pharmacokinetic profile [100,101]. Cardinali and Gong et al. have shown the beneficial effects of melatonin in several in vivo experimental models of neurodegeneration [102,103]. Recent results support the proof of using melatonin to improve cognitive function in AD. It is demonstrated that melatonin significantly diminished amyloid plaque formation in an animal model of AD (Tg2576 transgenic mice) [104]. In the animal model AD, neuroprotection provided by melatonin appears to be age-dependent. Peng et al. have reported that only in elderly mice (12–21 months old mice), melatonin revealed a cognition improvement after melatonin supplementation, compared with young mice (4-8 months old mice) [105]. Another study has also demonstrated this relationship, which has shown that only in old Tg2576 (APPswe) mice dosed melatonin (0.08 mg/day) showed a positive effect on cognitive function [106]. Furthermore, it has been reported that melatonin in all used dosages (2.5, 5, and 10 µM) significantly suppresses the procession of apoptosis in vitro through the regulation of apoptotic protein levels in ICR mice [103]. Additionally, melatonin plays a pivotal role in tauopathy, by reducing Tau protein phosphorylation through the regulation of GSK-3b and PP2A, consequently blocking the formation of the toxic aggregates [107]. 

Furthermore, it has been reported that melatonin inhibits the levels of mitochondrial damage and improves the viability of neurons [103]. Epidemiological studies have demonstrated that exposure to formaldehyde (FA) results in fatigue, sleep, headaches, anxiety, and in particular, cognitive disorders. Thus, FA-injected animals are an appropriate model for the study of cognitive impairment [108,109]. According to the results of animal experiments, gaseous FA (3 mg/m^3^), for 7 consecutive days of exposure, induces abnormal behaviors, such as depression, aggression, locomotor activity impairment, and spatial memory deficits. Notably, melatonin present in the brain is a powerful anti-oxidant protecting against neuronal death, and its decrease is observed in gaseous FA-exposed mice and AD patients [110]. 

Moreover, Mei et al. have reported, melatonin supplementation can alleviate brain oxidative stress, attenuate hippocampal structural damage, and restore gaseous FA exposure-induced cognitive decline [111]. That result is compatible with observation in a previous study where a liquid FA-injected rat model was used [112]. These data have confirmed that brain melatonin depletion contributes to FA exposure-related cognitive impairment in animal models. In addition, another study has found that melatonin alleviates age-related cognitive impairments and decreases the ROS production by mitochondria in the prefrontal cortex and hippocampus of aging rats [113]. 

#### 3.1.6. Sulforaphane

Sulforaphane (1-isothiocyanato-4-methylsulfinylbutane) is a compound within the isothiocyanate group of organosulfur compounds and is mainly found in cruciferous vegetables. Despite intensive research, the distribution of SFN and its metabolites in the tissues remains unclear. However, Clarke et al. Have shown that SFN penetrates the BBB. In mice, after oral administration, SFN was available in all tissues (brain, plasma, kidney, liver, colon, small intestine, prostate, and lung) after 2 and 6 h, while the highest concentration in the brain was reached after 2 h [114]. Several in vivo studies have demonstrated the effects of sulforaphane on cognition improvement in animal models of neurodegenerative diseases. Hou et al. have demonstrated that sulforaphane administration via intraperitoneal (5 mg/kg for 4 months) protected PS1V97L transgenic mice from cognitive deficits compared with control mice (mice no fed with sulforaphane). Furthermore, it has been demonstrated that sulforaphane inhibits Aβ aggregation, tau hyperphosphorylation, and oxidative stress in sulforaphane-fed PS1V97L transgenic mice, evaluated through GSH and MDA levels. Additionally, sulforaphane diminished the levels of pro-inflammatory cytokines TNF-α and IL-1β [115]. 

In addition, Wang et al. have reported the favorable effects of sulforaphane in AD rats. They demonstrated that sulforaphane, injected intraperitoneally at 5 mg/kg per day for 7 days, improved physical condition and spatial learning. Moreover, sulforaphane presumably inhibits depressive states, via the serotonergic system, by modulating both activities of the enzyme tryptophan hydroxylase, which is engaged in the metabolism of serotonin and as a transporter for serotonin. In the same study, it was been shown that in rat brains, treatment with sulforaphane diminished neuroinflammation and oxidative stress, respectively measured through decreased levels of MDA, TNF-α, and IL-1β as well as by an enhanced level of GSH [116].

In another study, Zhang et al. have reported that the effects of sulforaphane (25 mg/kg) administered orally in C57BL/6 mice with Alzheimer-like lesions improved cognitive and locomotor deficits evaluated by standard tests such as the Morris water maze and the open field test. Furthermore, it has been demonstrated that sulforaphane protects against the formation of Aβ plaques in the cortex and hippocampus, decreased by oxidative stress [117].

Studying the multiple sclerosis (MS) animal model, Li et al. have shown that the intraperitoneal injection with sulforaphane (50 mg/kg) inhibits the development and progression of the MS at female experimental autoimmune encephalomyelitis (EAE) C57Bl/6 mice induced subcutaneously with myelin oligodendrocyte glycoprotein peptide 35–55 (250 μg). In addition, they have reported that sulforaphane, by upregulated the Nrf2/ARE pathway, enhances the expression levels of nicotinamide adenine dinucleotide phosphate quinone oxidoreductase 1 (NQO-1) and heme oxygenase 1 (HO-1), leading to a reduction of oxidative stress. Moreover, sulforaphane silences the Th17-related inflammation initiated by the enhancement of the anti-inflammatory cytokine IL-10 response. Additionally, sulforaphane protects BBB permeability by reducing MMP-9 expression level and improving occludin and claudin-5 distribution [118].

#### 3.1.7. *N*-acetylcysteine 

Studies on animal models have reported that NAC shows the activity to effectively penetrate the BBB increasing the GSH levels in the brain [119,120]. NAC, regardless of the route of administration (orally, intravenously or inhaled) and dose, is well tolerated and safe. However, oral delivery is associated with rapid intestinal absorption and hepatic metabolism, with peak plasma concentrations observed after 1–2 h [121]. The biggest limitation of the use of NAC is the low bioavailability of its free form (<10%), thus the achieved plasma and tissue concentration is at a low level [122]. Cognitive deficits, including spatial working memory, short-term spatial memory, and long-term episodic memory, are observed in almost every age-dependent neurodegenerative disease. Otte et al. have shown a noticeable spatial learning decline in transgenic mice (G72Tg) and concomitant synaptic deficits, which are most presumably due to mitochondrial dysfunctions resulting in enhanced ROS accumulation in the brain. Furthermore, they have demonstrated that chronic oral supplementation of NAC, a natural precursor of GSH, enhances the antioxidant capacity and improves spatial learning in G72Tg mice [123].

Joy et al. have proved that an NAC supplementation (50 mg/kg or 100 mg/kg doses) during the progression of AD in an animal model is likely to prevent neuronal degeneration by reduction of the neurofibrillary degeneration in the form of tau accumulation [124]. Furthermore, it has been reported that the primary hippocampal neurons exposed to amyloid beta oligomers (AβOs) are presumably causative agents of AD, exhibiting irregular Ca^2+^ supply, mitochondrial dysfunction, and defective structural plasticity [125]. In turn, More et al. have noted that, in AβOs-injected rats, NAC administration for 3-weeks prevented spatial memory deficits and redox imbalance [126]. In another study, using two different injury models in two various species, it has been found that early post-injury treatment with NAC reduces the behavioral deficits related to traumatic brain injury [127].

All animal model studies described are summarized in Table 1 below.

### 3.2. Cohort Studies and Randomized Controlled Trials (RCTs)

Antioxidant phytochemicals have high potential to enhance cognitive functions in humans. Nevertheless, clinical trials are necessary to clearly define the safety, optimal doses, and durations of therapies. The activity of these compounds, well documented in pre-clinical studies, has not yet been unequivocally confirmed in clinical trials, as there are only a few studies involving humans.

#### 3.2.1. Baicalin

Clinical trials of baicalin treatment have focused on determining the safety and tolerability profile of this flavonoid. In a phase I RCTs, Li et al. investigated the pharmacodynamic properties, tolerability, and safety of baicalein and baicalin (7-O-glucuronide of baicalein) after a single dose (100–2800 mg) in 72 healthy volunteers. Urine, blood, and stool samples were peeled for 48 h at regular intervals. The pharmacokinetic profiles of these compounds were as follows: the maximum concentration of the compound after administration—0.75–3.5 h and 0.5–3 h, terminal half-life—1.90–15.01 and 4.22–10.80 h, for baicalein and baicalin, respectively. At the same time, the total plasma clearance was <1% for both compounds. No serious adverse events after oral administration of baicalein and baicalin were reported in this study. Only 11 volunteers reported mild side effects at higher doses, requiring no further treatment (e.g., abdominal distension, constipation, somnolence, and dizziness). In addition, no changes in electrocardiogram or blood pressure, as well as any hepato- and nephrotoxicity, were observed [128]. 

In turn, Pang et al. investigated the pharmacokinetics, tolerability, and safety of increasing doses of baicalein in 33 healthy volunteers. The duration of therapy was 10 days, with participants receiving a single dose (200, 400, or 800 mg) of baicalein chewable on the first day. After the single dose (48 h), subjects received a double dose (morning and evening) on days 3–9, and the last single dose on day 10. In a multiple-dose pharmacokinetic study, it was observed that the maximum concentration of baicalein was reached on day 8 of therapy. The dose proportionality constants were 0.922 (90% CI 0.0.650–1.195), whereas the accumulation rate had achieved 1.66–2.07. Only mild and moderate adverse effects were noted in this study, in both the study and control group, such as abdominal pain, constipation, abdominal distention, erythema, and alanine transaminase (ALT) increase [129]. 

Furthermore, the safety of baicalin has been also confirmed in a randomized, double-blind, placebo-controlled trial conducted by Hang et al. The inflammatory profile and lipid levels were studied in patients with rheumatoid arthritis and coronary artery disease. They observed that 12-week dosing at baicalin at a dose of 500 mg/day had a high safety profile and meaningfully improved lipid and inflammatory parameters compared with placebo: triglycerides (TG) (1.12 ± 0.36 vs. 1.87 ± 0.46 mmol/L), total cholesterol (TCh) (2.87 ± 1.23 vs. 3.22 ± 1.07 mmol/L), apolipoprotein (APO) (1.31 ± 0.41 vs. 1.23 ± 0.29 g/L), high-density lipoprotein (HDL) (1.38±0.41 vs. 1.16±0.32 mmol/L), low-density lipoprotein (LDL) (1.73 ± 0.52 vs. 2.42 ± 0.57), cardiotrophin-1 (CT-1) (42.9 ± 13.7 vs. 128.4 ± 24.3 ng/mL), and high-sensitivity C-reactive protein (hs-CRP) (1.64 ± 0.38 vs. 3.9 ± 1.4 mg/dL) [130]. 

Nevertheless, despite many animal studies, there is still a lack of clinical trials that would show the beneficial effect of baicalin on cognition in humans.

#### 3.2.2. Quercetin

The beneficial effects of quercetin supplementation demonstrated in in vitro studies and animal models have not been translated into human studies. In addition, low BBB penetration and the slight bioavailability of quercetin, due to intense intestinal absorption and the long half-life of its metabolites in vivo, is a substantial limitation of using quercetin in humans. For these reasons, there are only a few clinical studies involving the neuroprotective and cognitive enhancement effects of quercetin [131]. 

In a comparative analysis (n = 57), Olson et al. examined the effect of quercetin (at a dose of 2000 mg, which is the–equivalent to 200 mg caffeine) on mood and vigilance compared with 200 mg caffeine or a placebo. Compared with placebo, 45 min after administration, caffeine is shown to improve alertness, self-esteem significantly, and the number of stimuli detected, reduce overall mood disorder, fatigue, and shorten reaction time. There is no statistically significant difference between the effect of quercetin administration on cognition function, but the results were intermediate between the caffeine and placebo group. Thus, it has been suggested that quercetin in doses available in dietary supplements and the diet has no effect on cognitive functions in humans [132]. 

In turn, Broman-Fulks et al. in a large study (n = 941), assessed the impact of 12-week quercetin supplementation (at a dose of 500 mg/day or 1000 mg/day) on cognitive functions in adults, as well as on the improvement of deficits in the elderly (>60 years old). Unfortunately, this study did not demonstrate the neurorestorative effects of long-term quercetin supplementation. Furthermore, a high level of quercetin in plasma was also not associated with psychomotor speed, memory, attention, reaction time, and cognitive flexibility in the entire study population [133].

Due to the documented limitations of the bioavailability to the CNS after oral administration of quercetin in humans, how enzymatically modified isoquercitin (quercetin-3-O-glucoside) (EMIQ^®^) affects cognitive function, blood pressure, and endothelial function was investigated. The study group received EMIQ^®^, 17-times more bioavailable than quercetin aglycone (absorbable form in intestines), at a dose of 2 mg quercetin equivalent/kg. There was no observed effect of EMIQ^®^ on cognitive functions, oxidative stress parameters, blood pressure, or arterial stiffness. However, EMIQ^®^ positively impacted endothelial functions through NO-mediated vasorelaxant activity and the prevention of oxidant-induced endothelial dysfunction (the flow-mediated dilatation response 1.80, 95% CI 0.23-3.37, P = 0.025) [134].

#### 3.2.3. EGCG 

It is noteworthy that many RCTs on the neuroprotective action of EGCG have been transformed into clinical trials. For example, one RCT (n = 31) investigated the effect of EGCG (at a dose of 300 mg/day) compared with placebo on brain activity and mood self-esteem. It has been shown that EGCG increases EEG activity, including alpha, beta, and theta brainwaves, mainly in the midline frontal and central regions. Furthermore, in the EGCG-treated group, people were also more relaxed and attentive [135]. 

In contrast, another RCT (n = 27) noted that a single oral administration of EGCG (135 mg and 270 mg) might modulate cerebral blood flow parameters in healthy volunteers but without affecting mood and cognitive performance [136]. Therefore, Lovera et al. conducted a pilot phase I/II study to determine a safe dose of polyphenon E (decaffeinated green tea catechin blend containing 65% of EGCG) to be administered to MS patients. Moreover, they evaluated the neuroprotective effects of polyphenon E through the effect on N-acetyl aspartate (NAA) level in the brain and estimated a correlation between plasma EGCG concentration and NAA changes. The study included 10 (phase I) and 13 (phase II) patients, aged 18-60 years, with relapsing-remitting or secondary-progressive phases of MS. EGCG, at a dose of 800 mg per day (2 × 400 mg), increased brain NAA level in MS patients. Interestingly, an increase in plasma EGCG concentration by 1 ng/mL is associated with a 0.9% growth in the NAA level [137]. 

The hepatotoxic effect of EGCG (at a dose of 800–1200 mg/day) has been also noted in a randomized, double-blind, placebo-controlled trial (PROMESA), which has assessed the effect of this phytochemical on slowing progression of multiple system atrophy (MSA). Moreover, in this study, no beneficial effect of EGCG on MSA was observed [138]. Due to growing evidence of liver toxicity of EGCG, the European Food Safety Authority (EFSA), the Scientific Panel on Food Additives and Nutrient Sources added to Food (ANS) has expressed an opinion on the safety of catechins in green tea (mainly EGCG) derived from various sources, such as food, supplements, and infusions. Based on scientific data, doses of EGCG <800 mg/day are generally considered as safe. However, the adverse effects of EGCG on the liver reported in the study are probably due to idiosyncratic reactions. Thus, it is difficult to determine the hepatotoxic dose. Moreover, it has been observed that hepatotoxic effects frequently occur in people with a higher body mass index (BMI). For this reason, Younes et al. issued a recommendation to conduct studies on the dose-response of hepatotoxicity of green tea catechins and inter- and intraspecies variability [139]. 

Nevertheless, most of the studies were conducted in small groups of patients, and further multicenter RCTs involving a larger number of patients are needed to confirm the efficacy, safety, and tolerability of EGCG in patients. Due to the promising results of the multimodal approach and EGCG supplementation for cognition in people with Down’s syndrome [140], the PENSA study has been launched to evaluate the effectiveness of the multimodal approach to slowing cognitive decline (SCD) in people carrying the apolipoprotein E ε4 allele with SCD [141]. Approximately 200 patients are to be enrolled in the study, divided into four groups: (1) multimodal intervention (physical activity, cognitive training, dietary counseling, and social commitment) + EGCG (at a dose of 400 to 600 mg/day before meals); (2) multimodal intervention + placebo; (3) lifestyle recommendations + EGCG; and (4) lifestyle recommendations + placebo—the observation period will be 12 months [141].

#### 3.2.4. Propolis

Propolis has a potential cognition-enhancing effect, including improving concentration, attention, verbal memory, and information processing. Therefore, Zhu et al. have investigated the effect of propolis administration (in a dose of 830 mg) for 24 months on the cognitive functions of elderly adults living at high altitudes (n = 60, mean age 72.8 years). They observed that the placebo group developed MCI after 24 months, while the propolis group’s Mini-Mental State Examination (MMSE) scores improved. Moreover, pro-inflammatory cytokine (IL-1β and IL-6) levels were significantly decreased, while those in the control group increased. Similarly, the levels of transforming growth factor β1 (TGFβ1) after administration of propolis increased significantly, while they decreased in the placebo group. Moreover, the obtained MMSE values were inversely correlated with the level of IL-1β and positively with the level of TGFβ1 [142]. 

In turn, Asama et al. investigated the effect of oral propolis supplementation for 24 weeks on cognitive functions in elderly people (aged 60 to 79). They showed that verbal memory enhanced significantly compared with the placebo group in the study, and the levels of urea nitrogen, uric acid, creatinine, TCh, and LDL significantly improved in the propolis-supplemented group. Moreover, in the subgroup with a higher neurocognitive index, it was shown that propolis also augmented concentration, information processing speed, and attention complexity. Additionally, no side effects of its consumption have been shown [143]. Thus, it seems that it may be a helpful nutraceutical in adjuvant therapy for people with cognitive impairment.

#### 3.2.5. Melatonin

Melatonin is produced internally and secreted principally by the pineal gland, at night, under physiological conditions [144]; moreover, it is also ingested in the diet from many plant foods, such as cereals (wheat, barley, and oats), fruits (grapes, cherries, and strawberries), vegetables (tomatoes and peppers), legumes and seeds, nuts, pistachio, as well as medical herbs (*Scutellaria biacalensis* and *Hypericum perforatum*) [145]. According to available data, a safe dose of melatonin for humans depends on the emerging disorders; however, it often oscillates between 1 and 10 mg/kg daily [146]. Several RCTshave been conducted on the effect of melatonin supplementation in improving cognitive functions. Meta analyses including RCT results, assessing the impact of melatonin on sleep quality in patients with neurodegenerative diseases, have shown a significant improvement in sleep quality. However, no effect on the improvement of cognitive function has been noted [147,148]. In contrast, a meta-analysis of melatonin supplementation in the perioperative period for delirium in the elderly has shown that, in the group treated with melatonin, the incidence of delirium is significantly lower (OR 0.310, 95%CI 0.19–0.50, I^2^ = 0.000) [149]. Similarly, the RCTs determining the effect of melatonin on cognition in women with breast cancer undergoing chemotherapy have shown that melatonin (at a dose of 20 mg/day) significantly enhances executive functions, episodic memory, both immediate and delayed, verbal fluency, and recognition [150]. However, despite the beneficial effects of using melatonin, it has not been proven whether enhancing cognitive function is associated with melatonin as an immunomodulatory compound, endogenous antioxidant, or a chronobiotic drug (a compound capable of changing the phase of the circadian time system).

#### 3.2.6. Sulforaphane

Due to presence of sulforaphane in consumable food, sulforaphane is an attractive candidate for further human study. In addition, it has demonstrated promise in pre-clinical models of traumatic brain and spinal cord injury [151,152]. Recent studies have reported that the administration of sulforaphane leads to favorable outcomes on human brain disorders such as autism [153]. Heretofore, many RCTs investigating the pharmacokinetics and pharmacodynamics of sulforaphane have been conducted [154]. However, there is no conclusive evidence from RCTs about the neuroprotective action of sulforaphane. So far, no RCTs have been conducted on sulforaphane’s efficiency, safety, and tolerance in individuals with neurodegenerative disease. Nouchi et al. analyzed the effect of administration of sulforaphane alone and in combination with brain training for 12 weeks on cognition in the elderly population. The authors showed that sulforaphane, alone, caused a significant improvement in working memory and processing speed compared with placebos. Importantly, brain training alone also strengthened cognitive function, while the combined intervention did not cause any positive pro-health effects [155]. It has been reported that GSH levels in the hippocampus and cortex are decreased in AD patients. In a small pilot RCT, it was demonstrated that, after 7-day daily sulforaphane supplementation, plasma GSH concentrations were correlated with levels of GSH in the thalamus and cortex, suggesting a positive effect on short-term memory and higher cognitive processes [156]. Very promising evidence from pre-clinical studies led to the initiation of RCTs to use sulforaphane in the adjuvant treatment of AD [157].

#### 3.2.7. *N*-acetylcysteine (NAC)

The potential of NAC in promoting cognitive health and alleviating cognitive decline associated with dementia is widely described [158]. NAC is also used in therapies to counteract neurodegenerative and psychiatric diseases [159]. It has been demonstrated that the use of NAC in patients with schizophrenia results in an improvement in working memory, and it has been suggested that the effectiveness of NAC therapy requires more extended intervention (⩾24 weeks) [160]. For this reason, RCTs were designed to determine the applicability of NAC in the treatment of neurodegenerative diseases, including PD, AD, and MS. In RCTs conducted by Monti et al., it has been shown that the use of NAC, both 500 mg orally twice a day and weekly intravenous infusion over 3 months reduced Parkinson’s symptoms, as well as enhanced dopamine binding in the brain [161]. 

To date, no RCTs have been performed with NAC in treating dementia; however, some studies have showed cognitive improvement with nutraceuticals in which NAC was one of the leading ingredients [162,163,164]. In addition, it has been demonstrated that the nutraceutical have show promise for slowing the worsening of AD. A nutraceutical preparation containing: NAC, folic acid, B12, α-tocopherol, S-adenosylmethionine (SAM), and acetyl-L-carnitine improved behavioral/mood complications and cognitive performance of AD patients. Nevertheless, better efficacy was noted in patients with a less advanced course of the disease [165]. 

In one study, Monti et al. demonstrated that intravenous NAC, administered once weekly and 500 mg/2× day for two months in SM patients, enhanced cognition and attention and glucose metabolism in several regions of the brain [166]. In turn, one RCT pilot study showed that NAC (at a dose of 1250 mg/3-times per day) caused a lasting reduction in fatigue in patients with progressive MS [167]. However, acetylcysteine, besides its beneficial effects, can also cause side effects. Adverse effects of varying severity have been reported since its introduction into clinical use. These include nausea and vomiting, hot flushes, rash, angioedema, tachycardia, hypotension, and bronchospasm. Systemic hypersensitivity reactions are the most common complications of intravenous acetylcysteine intake, while vomiting and nausea are the most common complications of oral administration. The side effects are believed to depend on the NAC concentration and infusion rate and the duration of treatment. A higher incidence of side effects was reported in the female gender and those with allergies or asthma in the family history [168]. 

### 3.3. Limitations

Despite the promising results of preclinical studies, there may be many reasons for the failure of clinical trials of nutraceutical use in proteinopathies underlying neurodegenerative diseases. One possibility is the multimodality of the compounds used. Despite its apparent benefit, it may interfere with a given compound’s actual mechanism of action on a pathological protein, leading to difficulties in determining optimal clinical concentrations and pharmacokinetics. Moreover, in most cases, there is no clearly defined mechanism for inhibiting toxicity and protein aggregation. It cannot be excluded that this is only related to weakening the interprotein interactions through non-specific binding at the initial stages of aggregation. Furthermore, compounds containing catechol groups can undergo auto-redox reactions to form reactive carbonyl groups, which can then form Schiff bases with amino groups in the fibrils of proteins, leading to the cross-linking of proteins. Moreover, most nutraceuticals do not have a clearly defined binding site for amyloidogenic proteins [169]. Thus, the necessity of clinical trials is unquestionable, but further preclinical studies are also necessary to eliminate the limitations of the problem mentioned above. 

A subsequent possible explanation for the lack of benefit in clinical trials is that the tests have not lasted long enough. It may be impossible to demonstrate the benefits of antioxidant therapy over a few years if the treatment is trying to reverse the effects of decades of ongoing impaired cognition. Such intervention may be too late in the cognition process to make a difference in older adults [170]. Furthermore, when considering the adequate duration of therapy, it is essential to remember that early diagnoses of neurodegenerative diseases may also improve the efficacy of antioxidants in human studies. The sooner the diagnosis is made, the sooner the appropriate treatment and supplementation are implemented, which has a better chance of preventing cognitive impairment in the early stages of the disease [171].

It is crucial to remember that the lack of advantages seen in current clinical trials does not disprove the pivotal role of antioxidants in cognition improvement. Instead, these results force us to evaluate optimal and personalized antioxidant therapies, the “perfect” patients to study, and the appropriate clinical trial duration.

## 4. Associations between Dietary Patterns and Neurodegenerative Diseases

Numerous studies have shown the effect of various types of diet on the improvement of cognitive functions [172,173,174,175]. The most frequently mentioned of which are the Mediterranean diet (MD) and the Dietary Approach to Stop Hypertension (DASH) diet, as well as a combination of both nutritional patterns–the MIND diet [174,175]. 

### 4.1. Mediterranean Diet (MD)

General health condition is observed not so much by individual groups of products or individual behavior but by the general nutrition model. Due to the high content of vegetables and fruits, fish, nuts, legumes, and grains, relatively low consumption of dairy products and meat, low caloric content, and variety of foods, MD is considered one of the most health-promoting models of nutrition. The high content of compounds with antioxidant and anti-inflammatory properties seems to be crucial in the effectiveness of MD in cognitive disorders. MD has a well-documented pro-health potential in cardiovascular disorders [176,177,178], improving cerebral blood supply and oxygenation. An essential assumption of MD is the regular intake of fish, rich in PUFAs, and olive oil, which are a source of monounsaturated fatty acids (MUFA), as well as moderate alcohol consumption, which plays an essential role in the prevention of age-related cognitive impairment and dementia [175]. The effect of MD on cognition function has been well documented [179,180]. A meta-analysis by Singh et al. has shown that higher adherence to MD decreases the risk of MCI and the rate of MCI to AD progression. This study included five randomized cohort studies with a total of 8019 participants, aged 62–80 years, followed by 2.2–8 years. The highest tertile of MD adherence, compared with the lowest tertile, has reduced the risk of cognitive impairment by 33% (HR 0.67, 95% CI 0.55–0.81, *p* < 0.0001). Moreover, subjects without cognitive dysfunction following the MD have been related with a lower risk of developing MCI (HR 0.73, 95% CI 0.56-0.96, *p* < 0.05) and the occurrence of AD (HR 0.64, 95% CI 0.46–0.89, *p* < 0.01) [181]. Loughrey et al. have presented similar conclusions in the meta-analysis (15 cohort studies, n = 41,492; and 2 RCTs, n = 309 and n = 162). They have shown that MD improves global cognition and episodic memory but does not affect working or semantic memory in elderly adults (aged ≥ 50 years) [182]. Thus, MD may indirectly support cognitive functions, but more research is needed to demonstrate its direct effect.

### 4.2. Mediterranean-DASH Intervention for Neurodegenerative Delay (MIND)

Therefore, in 2015 based on the analysis of cohort studies, Morris introduced the Mediterranean-DASH Intervention for Neurodegenerative Delay (MIND) diet, a hybrid of MD and DASH and intended to have a beneficial effect on the age-related cognitive decline [183]. In the mentioned and the following study, Morris et al. have observed that high consumption of green leafy vegetables (due to the high anti-oxidant content: flavonoids, polyphenols, ascorbic acid, α-tocopherol, lutein, indoles, and isothiocyanates) and blueberries (rich in anthocyanins) enhances cognitive functions, including working, episodic, and semantic memories, visuospatial ability, and perceptual speed [183,184]. In another large cohort study (16,058 women, mean age 74.5 ± 2.3 years), Berendsen et al. have searched for a relationship between the long-term use of a nutritional model meeting the assumptions of the MIND diet and the rate of cognitive aging. They have performed the dietary assessment five times over 14 years and have determined cognitive function four times (over 6 years). They have shown that a higher MIND index is associated with the maintenance of better verbal memory in later years (multivariable-adjusted mean between highest and lowest quintiles 95% CI 0.01–0.07, *p* < 0.01), but not with the general behavior of cognitive function [185]. The results of the presented studies seem very promising, but to introduce general recommendations regarding the everyday use of the MIND diet, it is necessary to conduct further studies involving a larger number of participants.

## 5. Conclusions

The current knowledge suggests that an appropriate modification of diet or lifestyle reduces the cognitive decline. In this paper, we reviewed the available literature in terms of the use of natural ingredients accessible in the daily diet, the increased supply of which may contribute to the improvement of the quality of life, especially in the case of the elderly suffering from neurodegenerative disease by improving their cognitive function. However, while the results of pre-clinical studies have been encouraging, they have not been unequivocally supported by the RCTs results. Nevertheless, the low toxicity of phytocompounds, as well as the documented health-promoting properties of these compounds in various conditions, imply the necessity of further, well-designed research because anti-oxidant compounds may be a good form of adjuvant therapy for neurodegenerative diseases.

## Figures and Tables

**Table 1:** Natural antioxidants improving cognition in animal models of neurodegenerative diseases.

Antioxidant Compound	Study Design	Form of Application, Doses, and Duration	Principal Findings	Ref.
Baicalin	APP/PS1 transgenic mice (AD model)	intragastrically administration, 100 mg/kg once daily for 2 weeks	-reduced microglia activation -diminished neuronal apoptosis -reduced levels of pro-inflammatory cytokines	[40]
	adult ICR mice injected with aggregated Aβ1–42 protein into the hippocampus (AD model)	oral administration, 30, 50, and 100 mg/kg once daily for 2 weeks	-improved memory deficits -reduced glial cell activation -diminished the level of IL-6 and TNF-α	[48]
	adult male Wistar rats injected with a single dose of 65 mg/kg STZ (diabetes model)	intragastrically administration, 50, 100, and 200 mg/kg once daily for 7 weeks	-reversed cognitive impairment -enhanced neuronal survival	[51]
	adult male Wistar rats injected with aggregated Aβ1–42 protein into the hippocampus (AD model)	intragastric administration, 50 and 100 mg/kg once daily for 20 days	-attenuated neuronal apoptosis -diminished expression of the pro-apoptotic protein Bax, cytochrome c and caspase-3	[56]
Quercetin	adult male Wistar rats injected by 6-OHDA (PD model)	oral administration, 100, 200, and 300 mg/kg once daily for 2 weeks	-decreased AChE activity -improved memory and learning -enhanced neuron density -increased activity of scavenging enzymes: SOD, CAT, and GPx in the hippocampus	[63]
	APPswe/PS1dE9 transgenic mice (AD model)	oral administration, 40 mg/kg once daily for 16 weeks	-improved learning and recognition -reduced mitochondrial dysfunction	[67]
	C57BL/6 mice	oral administration, 60 mg/kg once daily for 16 weeks	-inhibited activation of protein phosphatase 2C α a -activated AMPK pathway -reduced the level of inflammatory biomarkers -diminished expression of Aβ converting enzyme 1 -improved cognitive functioning	[68]
	adult male Wistar rats injected with a single dose of 3 mg/kg STZ (AD model)	intraperitoneal administration, 40 and 80 mg/kg once daily for 12 days	-increased learning -ameliorated memory impairment	[71]
	C57BL/6J female	oral administration of quercetin-rich onion (Quergold) powder, 4 g/kg once daily for 4 and 10 weeks	-attenuated learning -improved memory recall	[72]
Epigallocatechin gallate	male C57/BL mice induced by *N*-methyl-4-phenyl-1,2,3,6-tetrahydropyridine (PD model)	oral administration, 2 and 10 mg/kg once daily for 10 days	-improved cognition decline -protective effect to neurons	[73]
	adult male Sprague–Dawley rats	intragastric administration, 100 mg/kg once daily for 4 weeks	-reduced the BBB permeability -improved learning and memory	[79]
Polyphenon E	male Wistar rats	oral administration (water consumption), polyphenon E extract at the concentration of 0.1% and 0.5% once daily for 26 weeks	-improved reference and working memory-related learning ability -lowered plasma concentrations of lipid peroxides	[81]
Pinocembrin	adult male Sprague–Dawley rats (transient global cerebral ischemia model)	intravenous administration, 5 and 10 mg/kg once daily for 14 days	-alleviated cognitive impairments -diminished neurological scores -declined neuronal loss in the hippocampus -decreased level of GFAP-positive cells in the hippocampal CA1 region	[88]
Pinocembrin	adult male ICR mice injected with a single dose of 50 mg/kg STZ (diabetes model)	oral administration, 50 mg/kg once daily for 10 days	-enhanced neuronal survival in the frontal cortex and hippocampal CA3 region	[89]
Pinobanksin	male Wistar rats (vascular dementia model)	oral administration, 5 and 10 mg/kg once daily for 5 weeks	-improved cognitive performance	[90]
Ethanolic extract of propolis	adult male Wistar rats injected with aggregated Aβ25-35 protein (AD model)	oral administration, 100, 200, and 300 mg/kg once daily for 21 days	-reversed cognitive impairment -decreased MDA level	[92]
Propolis	adult male Sprague–Dawley rats with seizure symptoms after kainic acid-injection (neurodegeneration model)	oral administration, 75 and 150 mg/kg once daily for 5 times at 12 h intervals	-attenuated NO, TNF-α, and caspase-3 levels -prevented neuronal loss in the brain -ameliorated seizures attacks	[93]
P-coumaric acid	juvenile male Sprague–Dawley rats	disposable oral administrtion of 30 mg/kg	-reduced oxidative stress -inhibited genotoxicity -exerted neuroprotection -improved cognitive decline	[94]
Caffeoylquinic acid	APP/PS2 double-transgenic mice	oral administration, specialist diet with 5-caffeoylquinic acid (5-CQA) at a concentration of 0.8% once daily for 6 months	-ameliorated cognitive decline and neuronal loss	[96]
Melatonin	male ICR mice injected with aggregated Aβ1–42 protein into the hippocampus (AD model)	intraperitoneal administration, 2.5, 5, and 10 mg/kg once daily for 14 days	-suppressed procession of apoptosis through the regulation of apoptotic protein levels	[103]
	Tg2576 transgenic mice (AD model)	oral administration (water consumption), melatonin solution at a concentration of 0.5 mg/mL once a daily for 4 months	-diminished amyloid plaque formation	[104]
	Tg2576 transgenic mice (AD model)	intraperitoneal administration, 10 mg/kg once daily for 12 months	-improved cognition functioning	[105]
	Tg2576 transgenic mice (AD model)	oral administration (water consumption), 2.6 mg/kg once daily for 4 months	-improved cognition functioning	[106]
	adult male Bal b/c mice exposed to gaseous FA (3mg/m^3^) for 7 consecutive days (AD model)	disposable intracerebroventricular injection of melatonin at concentration 100 µM	-alleviated brain oxidative stress -attenuated hippocampal structural damage -restored gaseous FA exposure-induced cognitive decline	[111]
	male Wistar rats	intraperitoneal administration, 10 mg/kg once daily for 28 days	-alleviated age-related cognitive impairments -decreased the ROS production by mitochondria in the prefrontal cortex and hippocampus	[113]
Sulforaphane	PS1V97L transgenic mice (AD model)	intraperitoneal administration, 5 mg/kg once daily for 4 months	-preserved from cognitive deficits -inhibited Aβ aggregation, tau hyperphosphorylation -diminished oxidative stress level	[115]
	dult male Sprague–Dawley rats injected intracerebroventricular with AβOs (AD model)	intraperitoneal administration, 5 mg/kg once daily for 4 7 days	-improved physical condition and spatial learning -inhibited depressive states -decreased levels of MDA, TNF-α, and IL-1β -enhanced GSH level	[116]
	C57/BL mice	oral administration (water consumption), 25 mg/kg once daily for 80 days	-improved cognitive and locomotor deficits -protected against the formation of Aβ plaques in the cortex and hippocampus	[117]
	Female C57BL/6 mice injected subcutaneously with 250 μg MOG35-55 peptide (EAE; MS model)	intraperitoneal administration, 50 mg/kg once daily for 22 days	-inhibited development and progression of the MS -enhanced expression levels of NQO-1 and HO-1 -protected BBB permeability by reducing MMP-9 expression level and improved occludin and claudin-5 distribution	[118]
*N*-acetylcysteine	G72/G30 transgenic mice (schizophrenia model)	oral administration (water consumption), 1 mg/mL once daily for 5 weeks	-enhanced the antioxidant capacity -improved spatial learning	[123]
	male albino Wistar rats injected with colchicine into the lateral ventricle stereotaxically (15 µg) (AD model)	intraperitoneal administration, 50 and 100 mg/kg for 7 days	-reduced neurofibrillary degeneration	[124]
	juvenile male Sprague–Dawley rats	oral administration (jelly with NAC) 200 mg/kg once daily for 21 days	-prevented spatial memory deficits and redox imbalance	[126]

## Data Availability

Not applicable.
